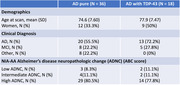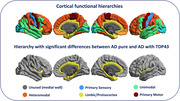# Impact of TDP‐43 co‐pathology on limbic cortical microstructure in Alzheimer's disease: evidence from an autopsy‐confirmed cohort with in vivo diffusion MRI

**DOI:** 10.1002/alz.094131

**Published:** 2025-01-09

**Authors:** Mario Torso, Gerard R Ridgway, Jamie Hardwidge, Ian Hardingham, Steven A Chance, National Alzheimer’s Coordinating Center, Alzheimer’s Disease Neuroimaging Initiative (ADNI)

**Affiliations:** ^1^ Oxford Brain Diagnostics, Oxford United Kingdom; ^2^ Oxford brain diagnostics, Oxford, England United Kingdom; ^3^ Oxford Brain Diagnostics, Oxford, Oxfordshire United Kingdom; ^4^ University of Washington, Seattle, WA USA; ^5^ University of Southern California, Los Angeles, CA USA

## Abstract

**Background:**

TDP‐43 (TAR DNA‐binding protein 43) is one of the most frequently observed co‐pathologies in Alzheimer's disease (AD). Recognizing the diversity of pathological features in individuals with AD, including the presence of TDP‐43, may lead to more personalized and effective treatment approaches. We investigate ante‐mortem cortical microstructural changes in MRI with subsequent autopsy confirmation of Alzheimer’s disease neuropathological changes (ADNC) with and without TDP‐43 comorbidity.

**Method:**

Participants with ADNC and ante‐mortem MRI were obtained from the National Alzheimer's Coordinating Center (NACC; n=46) and the Alzheimer's Disease Neuroimaging Initiative (ADNI; n=8). Participants were grouped based on TDP‐43 presence (36 AD “pure” and 18 AD with TDP‐43). Thal phase, CERAD neuritic plaque assessment, Braak NFT, and combined ABC score, were used as AD neuropathological hallmarks. Cases with TDP‐43 = Stage 2 were considered AD with TDP‐43. Structural and diffusion MRI were used to calculate cortical diffusivity measures in five functional macroregions: Primary Sensory, Unimodal, Limbic/Proisocortex, Heteromodal and Primary Motor (Ffytche & Wible 2014, PMID:24936089; Mesulam 1998, PMID:9648540). Cortical mean diffusivity and three minicolumn‐inspired cortical diffusivity measures were calculated: the angle between the radial minicolumnar direction and the principal diffusion direction (AngleR); the principal diffusion component parallel with the minicolumns (ParlPD), and the diffusion components perpendicular to the minicolumns (PerpPD+) (Torso et al. 2022, PMID:36281682). Groups were characterized using clinical, demographic, and neuropathological data (see table). Differences in diffusion metrics were tested adjusting for interval between MRI scan date and autopsy date, scanner manufacturer, diffusion b‐value, age and sex, with FDR correction (pFDR<0.05).

**Result:**

The Limbic/Proisocortex macroregion exhibited significantly higher AngleR (F1,36= 7.978; pFDR=0.04) in AD with TDP‐43 (AngleR mean 1.135) compared to the AD pure group (1.055).

**Conclusion:**

Diffusion MRI can detect differences in cortical microstructure of AD with TDP‐43 co‐pathology, suggesting more extensive cortical damage in AD with TDP‐43 comorbidity that involves Limbic cortical areas (cingulate, entorhinal, parahippocampal, and temporal pole). Consistent with previous findings regarding early cortical changes associated with amyloid deposition and neuroinflammation (Torso et al. 2022, PMID:36281682 Torso et al. 2323PMID:37794477), the diffusion‐derived metric AngleR appears particularly sensitive to subtle effects, such as the impact of TDP‐43 in addition to ADNC.